# Supplementary material for: Glassy adhesion dynamics govern transitions between sub-diffusive and super-diffusive cancer cell migration on viscoelastic substrates
Source: Nat Commun. 2026 Jan 13;17:978. doi: 10.1038/s41467-025-67709-1 (PMC12848116; doi:10.1038/s41467-025-67709-1)
Supplement: Supplementary file 1 — Supplementary Information [file 41467_2025_67709_MOESM1_ESM.pdf]

Glassy Adhesion Dynamics Govern Transitions Between Sub-Diffusive and Super-Diffusive Cancer Cell Migration on Viscoelastic Substrates

Vivek Sharma[1,2#], *Kolade Adebowale*[3,4,5#], *Ze Gong*[6#], *Ovijit Chaudhuri*[7,8*], *Vivek B. Shenoy*[1,2,9*]

[1] Center for Engineering Mechanobiology, University of Pennsylvania, PA, USA
[2] Department of Mechanical Engineering, University of Pennsylvania, PA, USA
[3] Shu Chien-Gene Lay Department of Bioengineering, University of California, San Diego, La Jolla, CA, 92093
[4] Program in Immunology, University of California, San Diego, La Jolla, CA, 92093
[5] Moores Cancer Center, University of California, San Diego, La Jolla, CA, 92093
[6] CAS Key Laboratory of Mechanical Behavior and Design of Materials, Department of Modern Mechanics, University of Science and Technology of China, Hefei, China
[7] Department of Mechanical Engineering, Stanford University, Stanford, CA, USA
[8] Chemistry, Engineering, and Medicine for Human Health (ChEM-H), Stanford University, Stanford, CA, USA.
[9] Department of Materials Science and Engineering, University of Pennsylvania, Philadelphia, PA, USA.

[#]Equal contribution
[*]Corresponding authors

Supplementary Information

Supplementary Note 1: Dissociation time constant distribution

In the main text, we argue that broad, glass-like distributions of adhesion lifetimes are essential for capturing the full spectrum of cell-substrate interactions that underlie anomalous migration. This first supplementary note therefore lays out the statistical form of the dissociation-time distribution we impose on clutches, explains the physical meaning of its two parameters (the glass coefficient β and the minimum timescale τ_0), and shows how these choices reproduce the heavy-tailed behaviour observed for real focal-adhesion proteins. We explain below how the glassy motor-clutch gets the long-tails, since the variance and higher moments derived below determine whether trapping events (β < 3) or rapid unbinding (β ≫ 3) dominate the subsequent migration dynamics.

The τ_{off} distribution is sampled from the following power law equation:

$$p(\tau_{off}) = \frac{|(\beta - 1)|}{\tau_{min}} \left(\frac{\tau_{off}}{\tau_{min}}\right)^{-\beta}$$

The parameter β primarily determines the "heaviness" of the distribution's tail, which is a key characteristic of glassy, heterogeneous systems like cell adhesion complexes where broad timescales are essential to capture both frequent short interactions and rare, prolonged interactions. This variance is sensitive to β alone, resulting in higher variability when β<3, which supports the long-tailed distribution that describes the complex temporal dynamics observed in biological adhesion processes.

In contrast, the parameter τ_{min} sets a lower bound on the dissociation timescale, representing the shortest possible time at which an adhesion bond can dissociate under typical cellular conditions. Physiologically, τ_{min} corresponds to the smallest timescale needed for initial, quick unbinding events—likely representing transient, weak interactions where bonds are formed but quickly broken under minimal force. By ensuring a minimum timescale, τ_{min} helps prevent unrealistic, infinitely fast dissociation in the model and reflects the natural constraints imposed by molecular interactions and energy barriers in cell adhesion complexes.

Variance of the distribution is its second moment.

$$\text{Distribution: } p(x) = \frac{\beta}{x_{min}} \cdot \left(\frac{x}{x_{min}}\right)^{-\beta}$$

Second moment:

$$V[x] = \int_{x_{min}}^{\infty} x^2 \cdot \frac{\beta}{x_{min}} \cdot \left(\frac{x}{x_{min}}\right)^{-\beta} dx$$

$$= \frac{\beta}{x_{min}^{1-\beta}} \cdot \int_{x_{min}}^{\infty} x^{2-\beta} dx$$

$$= \frac{\beta}{x_{min}^{1-\beta}} \cdot \left[x^{3-\beta}\right]_{x_{min}}^{\infty}$$

If $\beta \geq 3$, then the second moment converges and is finite.

If $\beta < 3$, then the variance is not finite and do not follow central limit theorem (CLT).

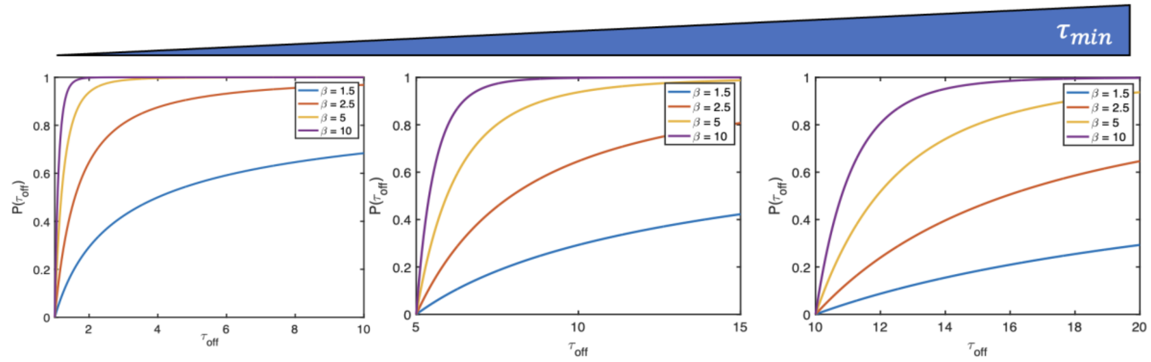

Supplementary Figure 1: Effect of long-tailed distribution parameters (τ_{min} and β) on the τ_{off} cumulative distribution. τ_{min} for the plots from left to right are 0.1, 5, and 10, respectively.

Further, the stochastic motor-clutch model can be simplified into a deterministic form by taking the mean behavior of all individual clutches and representing the entire ensemble by a single averaged clutch <.>. In this deterministic approximation, key clutch properties—such as binding probability, clutch force, substrate displacement, and retrograde flow velocity—are calculated using their mean values as shown below.

$$\frac{dP_b}{dt} = (1 - P_b)r_{on} - P_b < r_{off} >$$

$$< F_c > = k_c(< x_c > - < x_s >)$$

$$< x_s > = \frac{k_c n_c < x_c >}{k_s + n_c k_c}$$

$$< v_f > = v_u\left(1 - \frac{k_s < x_s >}{n_m F_m}\right)$$

$$\frac{d < x_c >}{dt} = (1 - P_b)\frac{d < x_s >}{dt} + P_b < v_f >$$

However, glassy motor-clutch model cannot be simplified into a deterministic framework. This is because the mean dissociation rate, $< r_{off} >$, is not valid due to the long-tailed distribution of adhesion lifetimes τ_{off}.

Supplementary Note 2: Effective off rate constant consisting of unfolding, unbinding and breaking:

The main text attributes glass-like adhesion lifetimes to two consecutive molecular events: a load-induced unfolding of clutch proteins such as talin or vinculin, followed by bond unbinding. This note defines in detail what each term means in protein-mechanics language, unfolding as a force-driven conformational change, unbinding as the physical separation of interacting partners, and clarifies that their combined outcome, dissociation, is what we treat as the effective off-rate for every clutch. By making these distinctions explicit, we link the microscopic steps of adhesion failure to the broad, heavy-tailed off-rate distribution introduced in Supplementary Note 1 and used throughout the glassy motor-clutch model.

Unfolding: Unfolding is the process where a protein loses its specific, folded three-dimensional structure (secondary and tertiary structures) due to applied force or environmental conditions, such as changes in pH or temperature. In adhesion dynamics, unfolding often refers to the stretching and unfolding of proteins like talin and vinculin under force. These proteins can act as molecular clutches: they bind to other proteins, and their unfolding under mechanical load allows cells to maintain traction forces while migrating.

Unbinding: Unbinding refers to the process where two proteins or molecular components that are physically interacting separate from each other without structural alteration to either. This process is primarily governed by the strength of the bonds or interactions (like hydrogen bonds, ionic interactions, and van der Waals forces) between the molecules. In focal adhesion complexes, unbinding might describe, for example, the separation of integrins from the extracellular matrix or other associated proteins.

Dissociation (off-rate): Dissociation in this context refers to the overall process by which a protein complex or interaction site completely separates, including any sequential steps required for this to occur. For a protein complex that is mechanically loaded, dissociation might involve both unfolding of structural domains (which occurs first) and unbinding of the final remaining contacts between proteins. Dissociation thus represents the culmination of sequential events where force may cause a protein to unfold before ultimately leading to unbinding and complete separation.

In summary, unbinding typically refers to the separation of interacting sites, unfolding describes a conformational change in response to force, and dissociation encompasses the entire sequence, often requiring both unbinding and unfolding in mechanotransducive processes. In adhesion mechanics, capturing the nuances of these events is essential, as each contributes uniquely to cellular responses and migration dynamics. Hence, we can write the effective dissociation rate as:

$$k_{off} = \frac{k_{unbind} * k_{unfold}}{k_{unbind} + k_{unfold}}$$

Now, an effective timescale can be written as:

$$\frac{1}{\tau_{off}} = \frac{\frac{1}{\tau_{unbind} * \tau_{unfold}}}{\frac{1}{\tau_{unbind}} + \frac{1}{\tau_{unfold}}} \tau_{off} = \tau_{unbind} + \tau_{unfold}$$

This relationship allows us to capture the stochastic nature of the unfolding and unbinding processes, which could each independently follow power-law distributions or we can treat unbinding as a constant timescale once proteins are unfolded. The key insight here is that in either case, the effective off-rate constant timescale distribution τ_{off} inherits power-law characteristics, displaying broad, glassy distributions with heavy tails.

This result is powerful because a power-law timescale distribution naturally aligns with the behavior observed in glassy systems. Specifically, the variance of τ_{off} can be rendered infinite for power-law exponents $\beta<3$, which promotes truly non-Gaussian, long-tailed distributions capable of capturing diverse adhesion behaviors. Therefore, modeling τ_{off} as a long-tailed power-law distribution enables us to encapsulate the heterogeneity present in adhesion dynamics, regardless of whether the stochasticity originates predominantly from unfolding or unbinding events. This ensures that our model accurately reflects the complex, glassy nature of adhesion formation and breakage dynamics in cell migration.

Supplementary Note 3: Solving the motor clutch model

This note presents the full mathematical formulation of the glassy motor-clutch model depicted schematically in Figure 2 of the main text. To solve each individual motor-clutch module, we adopt a Kinetic Monte Carlo approach, introduced in detail in our previous work [1]. Below, we outline the force-balance equations linking motors, clutches, and substrate, and then walk through the detailed algorithmic steps used to propagate the system forward in time.

Step 1: Initialize the model parameters based on the list provided in the table below (Table 1).

Step 2: Calculate ($r_{off,i}$) at current time t based on the current clutch forces ($F_{c,i}$) and the sampled off-rate (k_{off}) from the long-tailed distribution of (τ_{off}), and find the clutch bound/engaged probability ($P_{b,i}$) using the following master equation for clutches at all the ends.

$$\frac{dP_{b,i}}{dt} = (1 - P_{b,i})r_{on,i} - P_{b,i}r_{off,i}$$

Step 3: Choose a random number for each clutch. If ($P_{b,i} > rand$) the clutch is considered engaged, else it is considered disengaged.

Step 4: Use the updated number of engaged clutches to solve for the substrate displacement (x_s) using the following viscoelastic constitutive equation.

$$(k_a + k_l)\eta\frac{dx_s}{dt} + k_a k_l x_s = k_a F_s + \eta\frac{dF_s}{dt}$$

Step 5: Use force balance between myosin force, membrane resistance force, and adhesion force ($F_m + F_r = F_a$) in each + and – direction of X and Y direction to simplify the equilibrium equations into the following summation equations for two motor clutch modules in each direction:

Adhesion force balance in each +/- direction:

$$\sum_{i=1}^{n_c}(x_{c,i}^{+} + v_r^{-}\,dt - x_s)P_{b,i}^{+} = \sum_{i=1}^{n_c}(x_{c,i}^{+} + v_r^{+}\,dt - x_s)P_{b,i}^{-}$$

Rearranging the LHS using Hill's relation as outlined in the main text.

$$F_m\left(1 - \frac{v_r^{+} + v_r^{-}}{2v_m}\right) + F_r = k_c\sum_{i=1}^{n_c}(x_{c,i}^{+} + v_r^{+}\,dt - x_s)P_{b,i}^{+}$$

Step 6: Calculate the migration velocity and migration distance as:

$$v_m = \frac{v_r^{-} - v_r^{+}}{2}$$

$$d(t) = d(t-1) + v_m\,dt$$

$$F_r = k_m|\Delta x - \Delta y|$$

Step 7: Update the clutch forces($F_{c,i}$), myosin force (F_m) and membrane resistance force coupling the two dimensions (F_r) for the next simulation step ($t + dt$).

Step 8: Sample a new (τ_{off}) from the power law distribution to calculate (r_{off}) in the next cycle.

Variable	Parameter	Value	References
$\boldsymbol{k_a}$	Additional stiffness	0.1 – 10 pN/nm	Adjusted based on [2]
$\boldsymbol{k_l}$	Long-term stiffness	0.1 – 1 pN/nm	Adjusted based on [2]
$\boldsymbol{\eta}$	Viscosity	0.1-1000 pN-s/nm	Chosen to match the hydrogel relaxation timescales of experiments using ($\tau_s = \frac{\eta}{k_a}$)
$\boldsymbol{n_m}$	Myosin motor number (on one module)	200	Adjusted based on [3, 4]
$\boldsymbol{n_c}$	Clutch number (on one module)	200	Adjusted based on [3, 4]
$\boldsymbol{F_m}$	Myosin force	2 pN	Adjusted based on [3-5]
$\boldsymbol{F_b}$	Characteristic clutch breakage force	2 pN	Adjusted based on [3-5]
$\boldsymbol{r_{on}}$	Association rate	1 s^{-1}	Adjusted based on [3-5]
$\boldsymbol{\tau_{min}}$	Dissociation rate time constant distribution parameter	$1s^{-1}$	Chosen as a reference value of dissociation rate observed at zero force and as a parameter to modulate the power law in this model [3-5]
$\boldsymbol{\beta}$	Glassy coefficient	2.5	Chosen to follow a non-gaussian distribution SI note 1 and fine-tuned for the model as shown in Fig. 2E
$\boldsymbol{k_c}$	Clutch stiffness	5 pN/nm	Adjusted based on [3-5]
$\boldsymbol{v_u}$	Unloaded retrograde flow velocity	120 nm/s	Adjusted based on [3-5]
$\boldsymbol{v_p}$	Polymerization velocity	120 nm/s	Adjusted based on [3-5]

Supplementary Note 4: Model predicts diffusive migration mode on wide range of substrate parameters:

With an understanding of the hierarchy of timescales, we use our model to predict migration modes across a wide range of substrate parameters. Changing the substrate parameters (k_a: $additional\ stiffness, k_l$: $long-term\ stiffness, \eta$: $viscosity$) can affect the previously discussed timescales (τ_s, τ_l). Here, we use the glassy motor clutch model to obtain comprehensive phase diagrams of to show how the diffusivity exponent depends on the mechanical properties of the substrate. We choose β = 1.5 to capture the glassy dynamics resulting from the long tail distribution of τ_{off}, as increasing β results in a fast-decaying tail, making it incapable of capturing differences in trap time and step size distribution tailed-ness with changing viscous properties. Consequently, the model cannot capture both sub- and super-diffusive migration modes, regardless of viscosity changes.

We find that viscosity affects cell migration in distinct ways depending on the elastic properties (k_a, k_l), with the most significant impact at stiffnesses (k_a~$1kPa$). Stress relaxation is governed by $\sigma = k_l + k_a e^{-k_a t/\eta}$, for very small k_a (~0.1 kPa), and the time response is dampened due to the small coefficient multiplying the second term, which minimizes the effect of viscosity. In this case cells are unable to be trapped due to unstable clutches, leading to continuous migration steps and super-diffusive behavior. As k_a increases, the effect of viscosity becomes more pronounced. For $k_a \cong 0.2\ kPa$, a small region in the phase diagram starts to show sub-diffusive migration. With further increase in $k_a \cong 1\ kPa$, corresponding to the stiffnesses of our experimental alginate substrates, we observe that higher viscosities result in sub-diffusive migration, while lower viscosities lead to super-diffusive migration. At this intermediate level of "additional" stiffness, the force transmission is optimal, allowing clutches to remain trapped for longer on slow-relaxing substrates. Conversely, on fast-relaxing substrates, the rapid decrease in overall stiffness destabilizes clutches, inhibiting trapping and resulting in longer migration steps characteristic of super-diffusive migration. As shown in SI Fig. 2, traversing the vertical line along increasing viscosity, the diffusivity decreases from $\alpha \cong 1.4$ to $\alpha \cong 0.6$, corresponding to the range observed in our experiments when cells were seeded on fast- vs. slow-relaxing substrates. For a further increase in k_a (~10 kPa), viscosity effects diminish, and cells exhibit super-diffusivity for all parameter ranges due to rapid load and fail cycles that prevent trapping. Our previous experimental work also shows that viscosity only plays a role on soft substrates (k_a~$1\ kPa$) and cells on stiff substrates show no viscosity dependent effects [2]. Thus, sub-diffusion is only observed at higher viscosities on substrates with a stiffness of order of 1 kPa, whereas lower viscous properties and higher stiffness regimes predominantly results in super-diffusion. We next examine the role of

contractile forces in sustaining super-diffusive migration and how the impairment of contractility alters diffusivity.

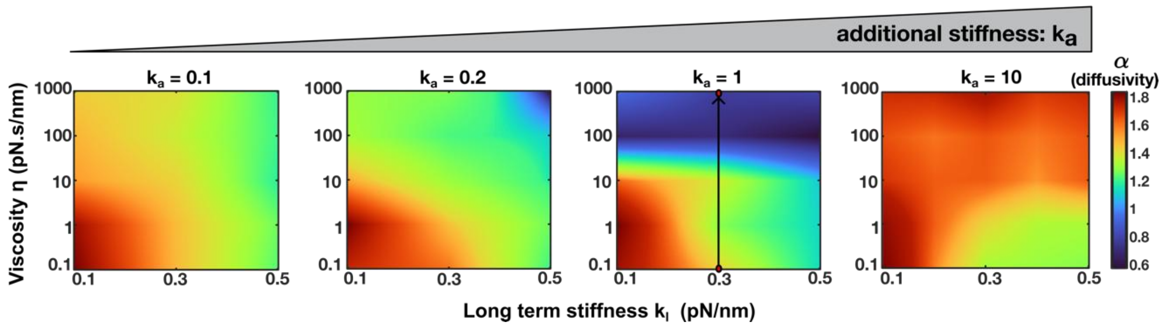

Supplementary Figure 2: Migration diffusivity on wide range of substrate parameters.

Supplementary Note 5: Substrate relaxation and creep timescales

In the standard linear solid (SLS) model, which consists of a spring-dashpot-spring assembly, both the stress relaxation and creep responses offer insights into the viscoelastic properties of a material under different loading conditions. The stress relaxation timescale $\tau_{relax} = \frac{\eta}{k_a}$ captures how stress dissipates under a constant strain, while the creep timescale $\tau_{creep} = \frac{\eta(k_a+k_l)}{k_a.k_l}$ represents how the material progressively deforms over time when a constant load is applied. Here, η is the viscosity of the dashpot, and k_l and k_a are the long-term and additional stiffness respectively.

Despite these definitions, both timescales are closely related and generally fall within the same order of magnitude (SI Fig. 3). In real experiments, materials often exhibit both relaxation and creep behaviors simultaneously, meaning either timescale can provide a

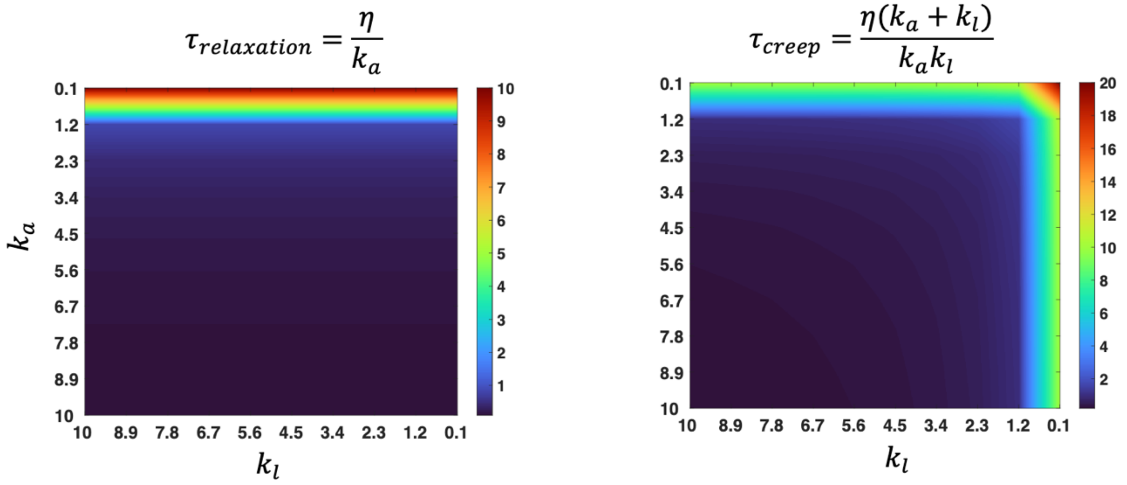

Supplementary Figure 3: Creep vs relaxation timescales as the substrate parameter changes.

valid approximation of the material's viscoelastic response. For simplicity in this work, we adopt the relaxation timescale, $\tau_s = \tau_{relax} = \frac{\eta}{k_a}$, given that rheology measurement on the alginate hydrogels measured stress relaxation timescale. This choice allows us to align our modeling with empirical observations, ensuring consistency with experimental conditions that focus on stress relaxation measurements.

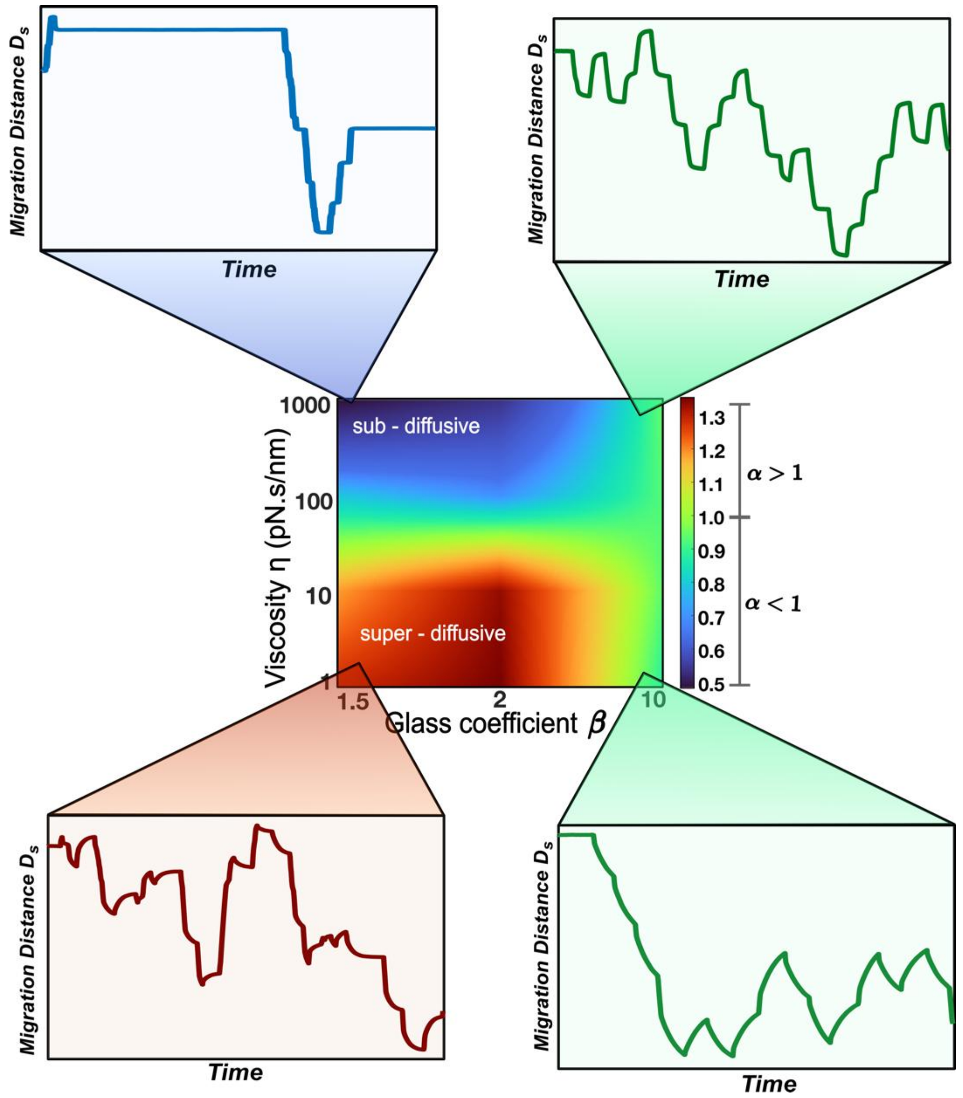

Supplementary Figure 4: Migration trajectories for glassy vs non-glassy model. Left represents migration trajectories for both fast (bottom) and slow (top) when glass coefficient value is small. Right side represents corresponding cases when glassiness is removed, and we see a periodic migration pattern for low and high viscosities.

NEW DATA

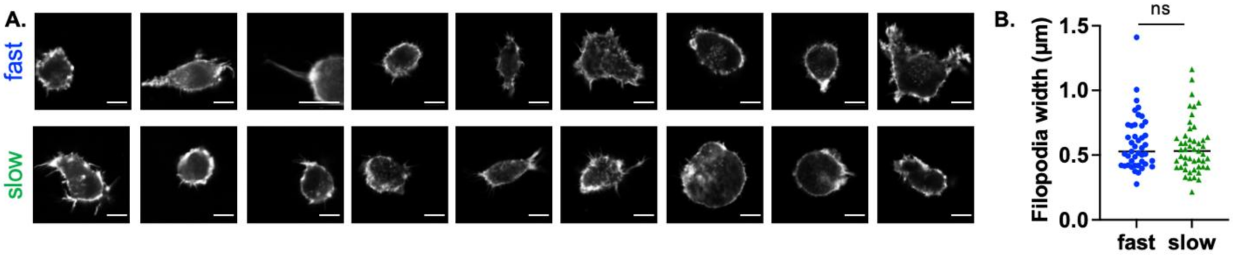

Supplementary Figure 5: A. Representative images showing filopodia morphology. **B.** Quantification of filopodia width from cells on fast and slow relaxing hydrogels. **A.** Scale bar: 10 μm. B. Kolmogorov-Smirnov test, ns: 0.6663, n > 43, N = 3.

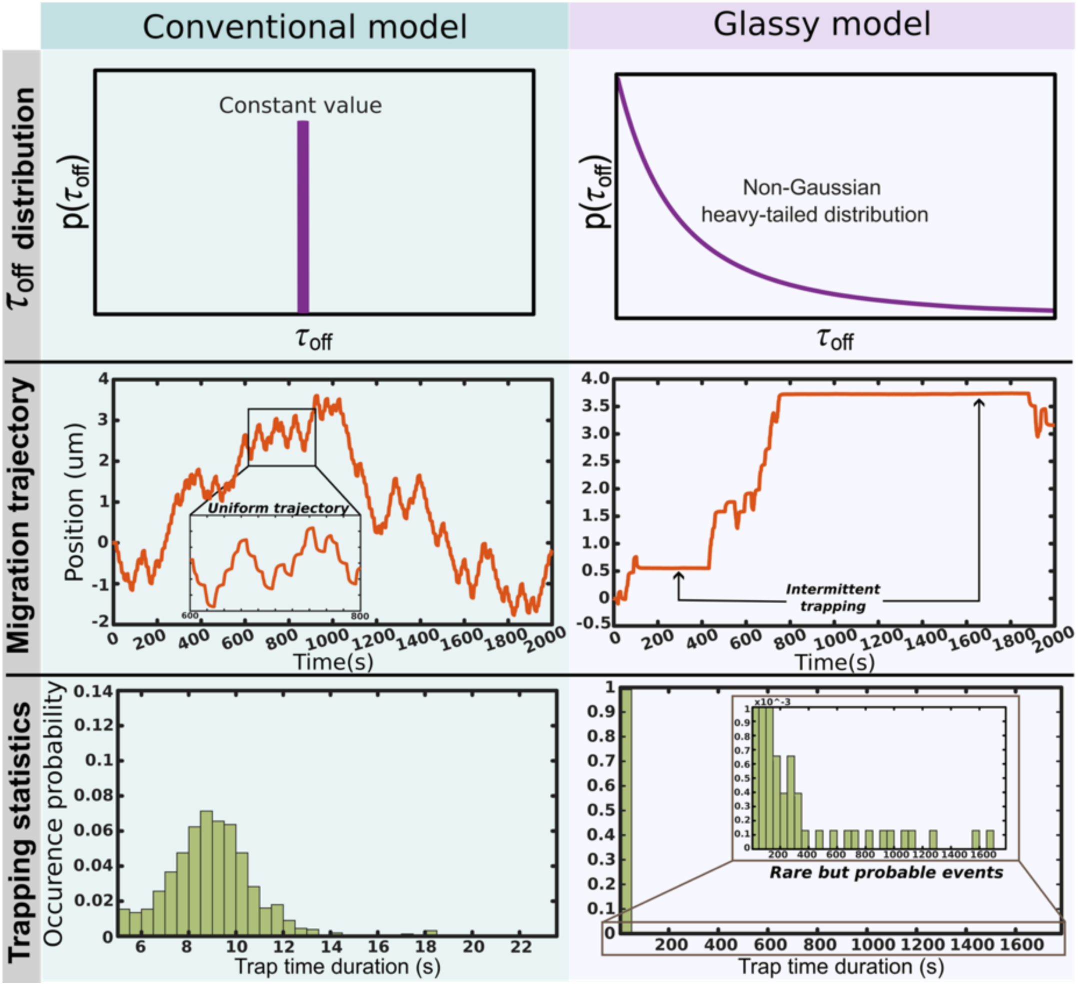

Supplementary Figure 6: Comparison of migration dynamics in conventional and glassy motor–clutch models. The conventional model (left) with constant τ_{off} produces uniform, Gaussian trajectories with narrowly distributed trapping times. In contrast, the glassy model (right) with a heavy-tailed τ_{off} distribution yields intermittent trapping and non-Gaussian trapping-time statistics with rare, long-lived events.

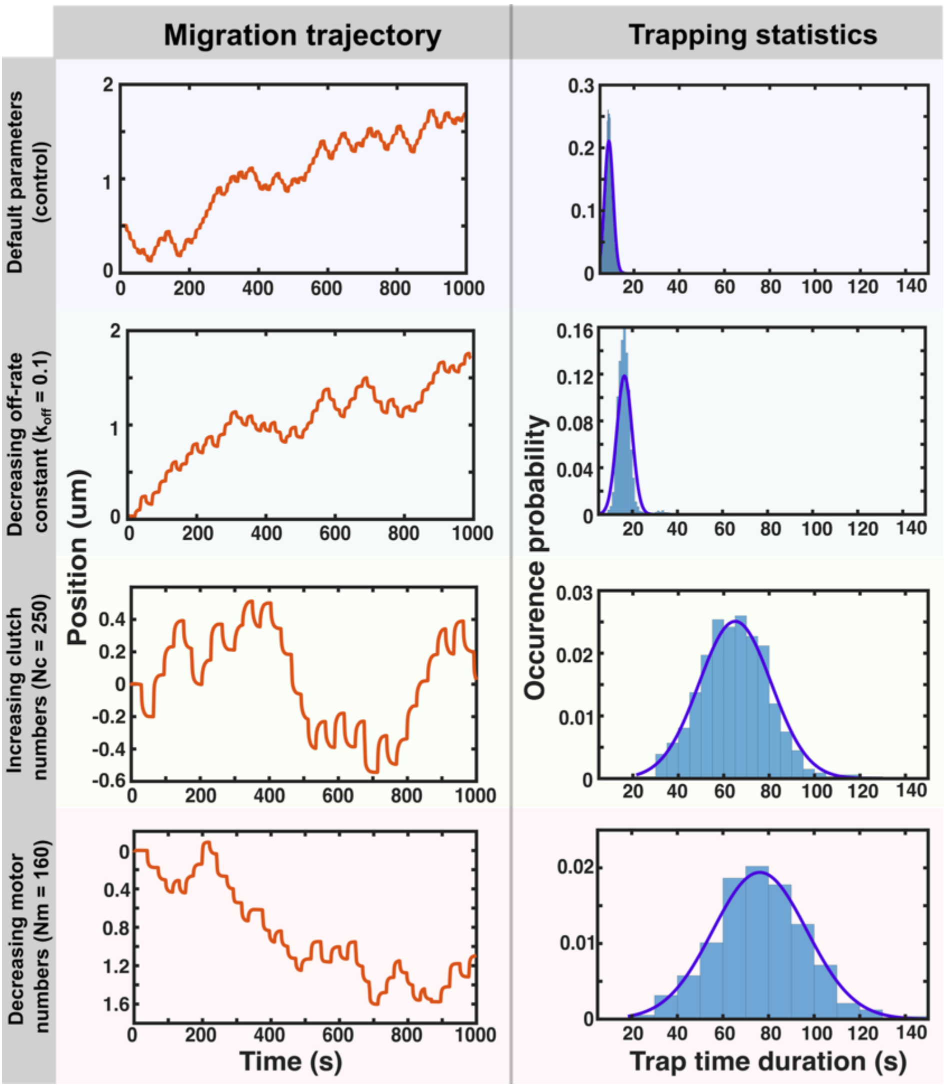

Supplementary Figure 7: No change in parameter in conventional models can produce anomalous intermittent trapping. Left: Migration trajectories for different parameter changes are plotted. **Right:** Histogram of trapping times for 50 trajectories shows a Gaussian distribution (blue line).

References:

1. Adebowale, K., Z. Gong, J.C. Hou, K.M. Wisdom, D. Garbett, H.-P. Lee, S. Nam, T. Meyer, D.J. Odde, V.B. Shenoy, and O. Chaudhuri, *Enhanced substrate stress relaxation promotes filopodia-mediated cell migration.* Nature Materials, 2021. **20**(9): p. 1290-1299.
2. Gong, Z., S.E. Szczesny, S.R. Caliari, E.E. Charrier, O. Chaudhuri, X. Cao, Y. Lin, R.L. Mauck, P.A. Janmey, J.A. Burdick, and V.B. Shenoy, *Matching material and cellular timescales maximizes cell spreading on viscoelastic substrates.* Proc Natl Acad Sci U S A, 2018. **115**(12): p. E2686-E2695.
3. Bangasser, B.L., G.A. Shamsan, C.E. Chan, K.N. Opoku, E. Tuzel, B.W. Schlichtmann, J.A. Kasim, B.J. Fuller, B.R. McCullough, S.S. Rosenfeld, and D.J. Odde, *Shifting the optimal stiffness for cell migration.* Nat Commun, 2017. **8**: p. 15313.
4. Chan, C.E. and D.J. Odde, *Traction dynamics of filopodia on compliant substrates.* Science, 2008. **322**(5908): p. 1687-91.
5. Bangasser, B.L. and D.J. Odde, *Master equation-based analysis of a motor-clutch model for cell traction force.* Cell Mol Bioeng, 2013. **6**(4): p. 449-459.
